# Supplementary material for: The Polish Society of Gynecological Oncology Guidelines for the Diagnosis and Treatment of Endometrial Carcinoma (2023)
Source: J Clin Med. 2023 Feb 13;12(4):1480. doi: 10.3390/jcm12041480 (PMC9959576; doi:10.3390/jcm12041480)
Supplement: Supplementary file 1 [file jcm-12-01480-s001.zip › File S1.pdf]

File S1. **ProMisE classifier and comprehensive endometrial carcinoma diagnosis algorithm NGS+IHC**

Promise

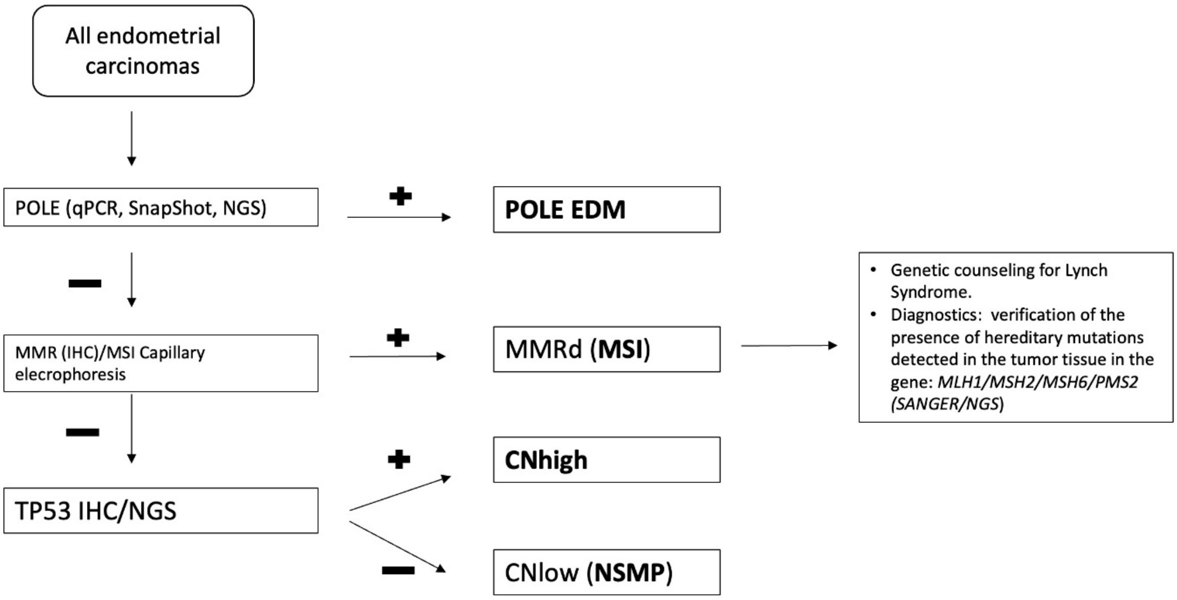

Comprehensive endometrial carcinoma diagnosis algorithm

- Parallel IHC evaluation of TP53 and MMRd protein expression (MLH1, MSH2, MSH6, PMS2) and panel sequencing by NGS (*MLH1, MSH2, MSH6, PMS2, TP53, POLE, POLD1, BRCA1, BRCA2, CTNNB1* (β-catenin) exon 3,

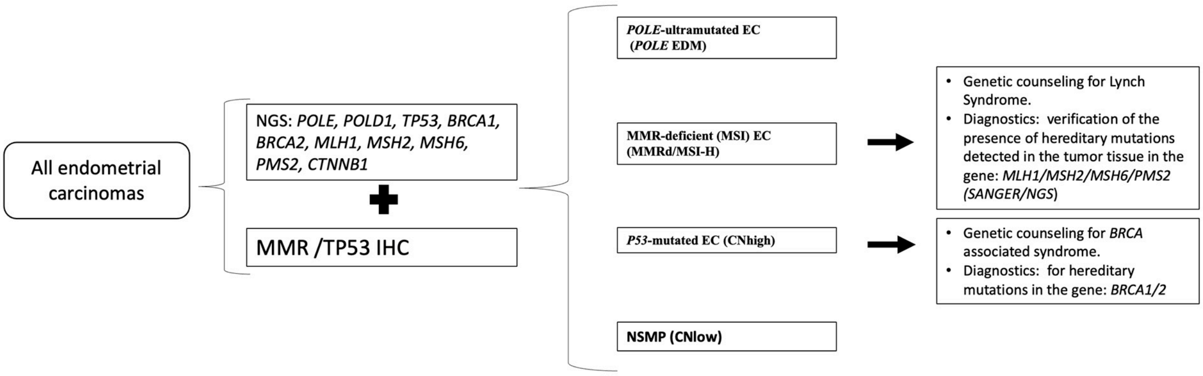

- The use of this algorithm allows to classify the patient to the molecular type. In addition, it improves further diagnostics towards hereditary syndromes (Lynch syndrome and BRCA1 / 2 mutation-related syndromes)
