# Supplementary material for: The Polish Society of Gynecological Oncology Guidelines for the Diagnosis and Treatment of Endometrial Carcinoma (2023)
Source: J Clin Med. 2023 Feb 13;12(4):1480. doi: 10.3390/jcm12041480 (PMC9959576; doi:10.3390/jcm12041480)
Supplement: Supplementary file 1 [file jcm-12-01480-s001.zip › File S2.pdf]

**File S2: Postoperative final report**

As recommended by PTP (Polish Society of Pathology) (Pathology: standards and examples of good practice and differential diagnosis. Guidelines for pathology departments/laboratories. Polish Ministry of Health) postoperative final report (radical hysterectomy for carcinoma of the endometrium) should include at least:

- macroscopic description of surgical specimen and identified pathological changes
- microscopic diagnosis (histological type of the tumour and ICD-O code) *
- grading
- lymph node status (number of lymph nodes and number of metastatic nodes)
- TNM staging
- assessment of lymph- vascular space invasion (LVSI)

PTGO recommends supplementing the final report with additional clinically relevant prognostic and predictive factors:

- molecular subtype of endometrial cancer, at least the ProMisE version (if it was not performed before surgery)
- ER/PR receptor status (for low-grade endometroid carcinoma)
- HER2 receptor status (for serous carcinoma) **
- type of involvement/invasion of lymph spaces: focal or substantial LVSI ***
- ultrastaging of lymph nodes when sentinel node procedure is used instead of systematic lymphadenectomy

* Reliable assessment of histological type of cancer, especially in the case of high-grade tumours requires- apart from routine hematoxylin and eosin staining- performing several additional tests (immunohistochemistry), including steroid receptors (ER, PR), vimentin, proteins: P16 and P53 and antigens: PAX8, WT1, GATA3, AMACR

** Determination of HER2 gene status should be preceded by a differential diagnosis of serous carcinoma, endometroid carcinoma, clear cell adenocarcinoma and mesonephric carcinoma- as a rule, it requires performing above mentioned immunohistochemical tests

*** Pathologist evaluating LVSI must remember about several changes imitating the involvement of lymphatic vessels, such as: (a) displacement of neoplastic tissue into the gaps in the uterine muscle, most often as a result of manipulation during surgery or initial assessment of unfixed tissue specimen (b) collapse/contraction of the stroma surrounding the tumour during formalin fixation.
