# Supplementary material for: The Polish Society of Gynecological Oncology Guidelines for the Diagnosis and Treatment of Endometrial Carcinoma (2023)
Source: J Clin Med. 2023 Feb 13;12(4):1480. doi: 10.3390/jcm12041480 (PMC9959576; doi:10.3390/jcm12041480)
Supplement: Supplementary file 1 [file jcm-12-01480-s001.zip › File S4.pdf]

File S4

Carcinoma of the endometrium– Determination of gene and HER2 receptor status

Recent studies have shown a significant role in determining the status of the HER2 gene and its protein product in serous endometrial cancer. In the clinical trials conducted so far, the qualification for personalised treatment of patients with serous cancer was mainly based on the evaluation of HER2 receptor expression in an immunohistochemical test. The reaction of high intensity (3+) found in more than 30% of cancer cells is considered positive. When assessing it, it should be remembered that the morphology of the response to the presence of the HER2 receptor in serous endometrial cancer is slightly different from that observed in breast cancer. First, in endometrial cancer cells, in most (about 75%) cases, a "U-shaped" basolateral membrane staining or as a immunostaining only of the lateral parts of the cell.

Secondly, the result of the immunohistochemical test in more than half of the cases with positive expression of the HER2 receptor is heterogeneous - next to the fields with a clearly positive reaction (HER2-3+), fragments of the tumor with a less intense reaction (at most HER2-2 +) are found. In cases where the result of the immunohistochemical test is questionable (HER2-2+), it is recommended to assess the status of the HER2 gene using the FISH technique (figure 1)

The condition for obtaining a reliable result of the immunohistochemical test as well as the *HER2* gene status test using the FISH technique is the correct fixation of the biopsy and postoperative material in a 10% buffered formalin solution at pH 7.2 - 7.4 for a period of 6-72 hours. A pathologist assessing the tissue material should remember, that eligible for the HER2 receptor testing for personalised treatment are patients with serous carcinoma or mixed carcinoma with serous carcinoma component. In the latter case, the fragment of the tissue section (and the corresponding fragment of the paraffin block) containing the greatest amount of serous carcinoma should be selected for immunohistochemical and/or molecular testing.

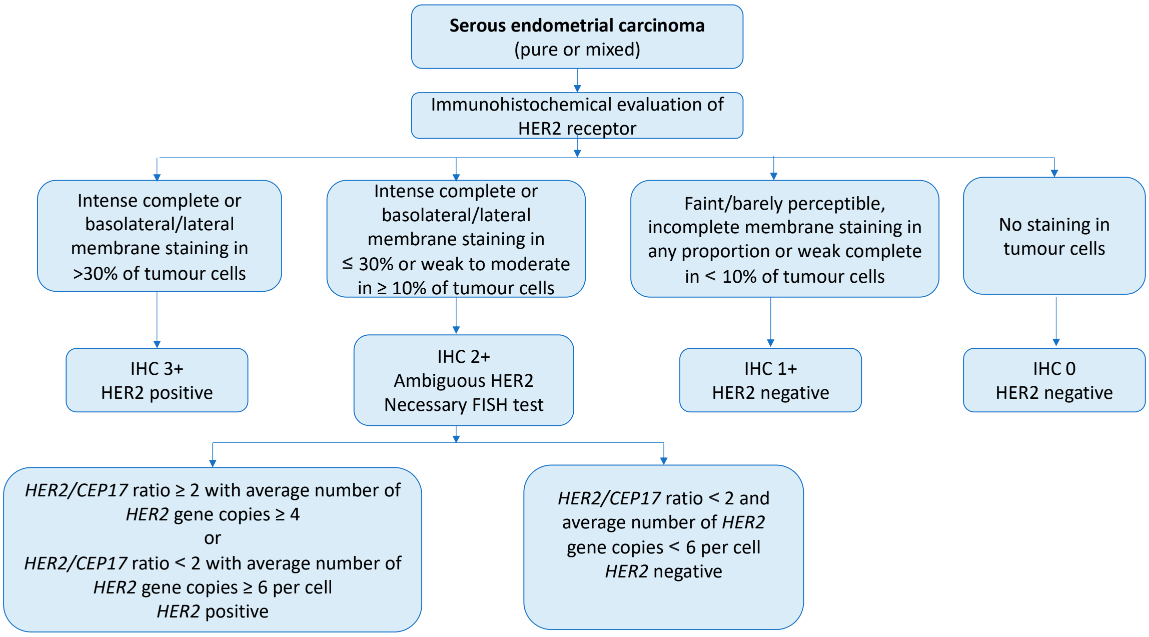

Figure

Bibliography:

[1_Supp4] Buza N.: HER2 testing in endometrial serous carcinoma. Time for standarized pathology practice to meet the clinical demand. Arch Pathol Lab Med. 2021; 145:687-691

[2_Supp4] Buza N.: HER2 Testing and Reporting in Endometrial Serous Carcinoma: Practical Recommendations for HER2 Immunohistochemistry and Fluorescent In Situ Hybridization: Proceedings of the ISGyP Companion Society Session at the 2020 USCAP Annual Meeting. Int J Gynecol Pathol 2020, 40:17-23
