# Supplementary figures and images for: The Polish Society of Gynecological Oncology Guidelines for the Diagnosis and Treatment of Endometrial Carcinoma (2023)
Source: J Clin Med. 2023 Feb 13;12(4):1480. doi: 10.3390/jcm12041480 (PMC9959576; doi:10.3390/jcm12041480)

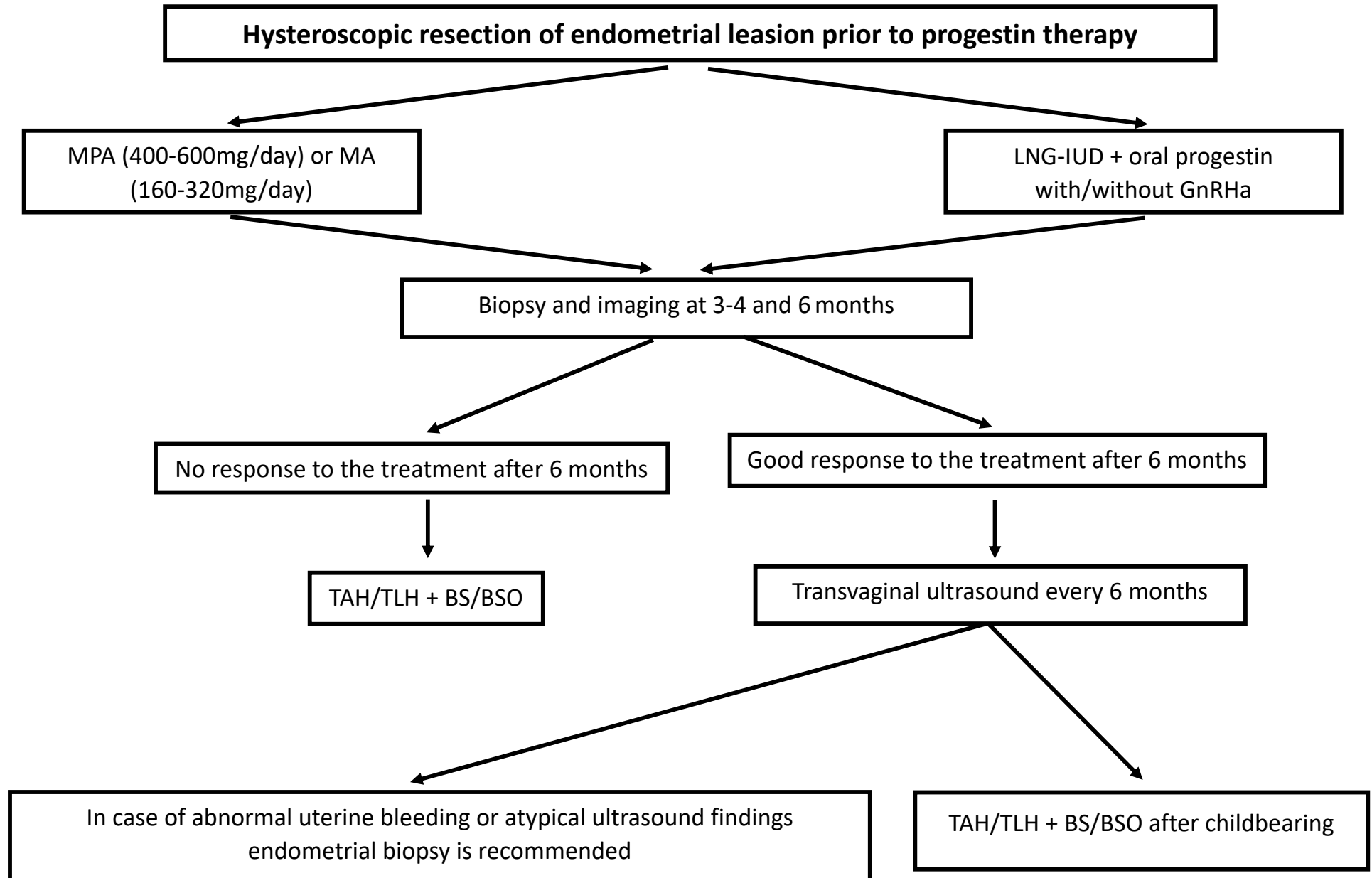

Supplement: Supplementary file 1 [file jcm-12-01480-s001.zip › File S3.pdf]
